# The regulatory status of health apps that employ gamification

**DOI:** 10.1038/s41598-024-71808-2

**Published:** 2024-09-09

**Authors:** Oscar Freyer, Kamil J. Wrona, Quentin de Snoeck, Moritz Hofmann, Tom Melvin, Ashley Stratton-Powell, Paul Wicks, Acacia C. Parks, Stephen Gilbert

**Affiliations:** 1 Else Kröner Fresenius Center for Digital Health, TUD Dresden University of Technology, Dresden, Germany; 2 WhalesDontFly H&F GmbH, Berlin, Germany; 3 Bielefeld University of Applied Sciences and Arts, Bielefeld, Germany; 4 Therapixel, Nice, France; 5 School of Medicine, Trinity College, University of Dublin, Dublin, Ireland; 6 RQM+, Altrincham, Cheshire, UK; 7 Wicks Digital Health, Advantage House, Stowe Court, Lichfield, UK; 8 Liquid Amber, Cortland, OH USA

**Keywords:** Health policy, Translational research

## Abstract

Smartphone applications are one of the main delivery modalities in digital health. Many of these mHealth apps use gamification to engage users, improve user experience, and achieve better health outcomes. Yet, it remains unclear whether gamified approaches help to deliver effective, safe, and clinically beneficial products to users. This study examines the compliance of 69 gamified mHealth apps with the EU Medical Device Regulation and assesses the specific risks arising from the gamified nature of these apps. Of the identified apps, 32 (46.4%) were considered non-medical devices; seven (10.1%) were already cleared/approved by the regulatory authorities, and 31 (44.9%) apps were assessed as likely non-compliant or potentially non-compliant with regulatory requirements. These applications and one approved application were assessed as on the market without the required regulatory approvals. According to our analysis, a higher proportion of these apps would be classified as medical devices in the US. The level of risk posed by gamification remains ambiguous. While most apps showed only a weak link between the degree of gamification and potential risks, this link was stronger for those apps with a high degree of gamification or an immersive game experience.

## Introduction

Smartphone applications (i.e., mHealth apps) are one of the main delivery modalities in digital health^1^. As with other end-user-facing apps, user engagement is critical to the efficacy of these healthcare apps^2,3^. One strategy to augment engagement and enhance user experience in these apps involves integrating elements that have demonstrated efficacy in digital games^4–7^.

**Fig. 1:**
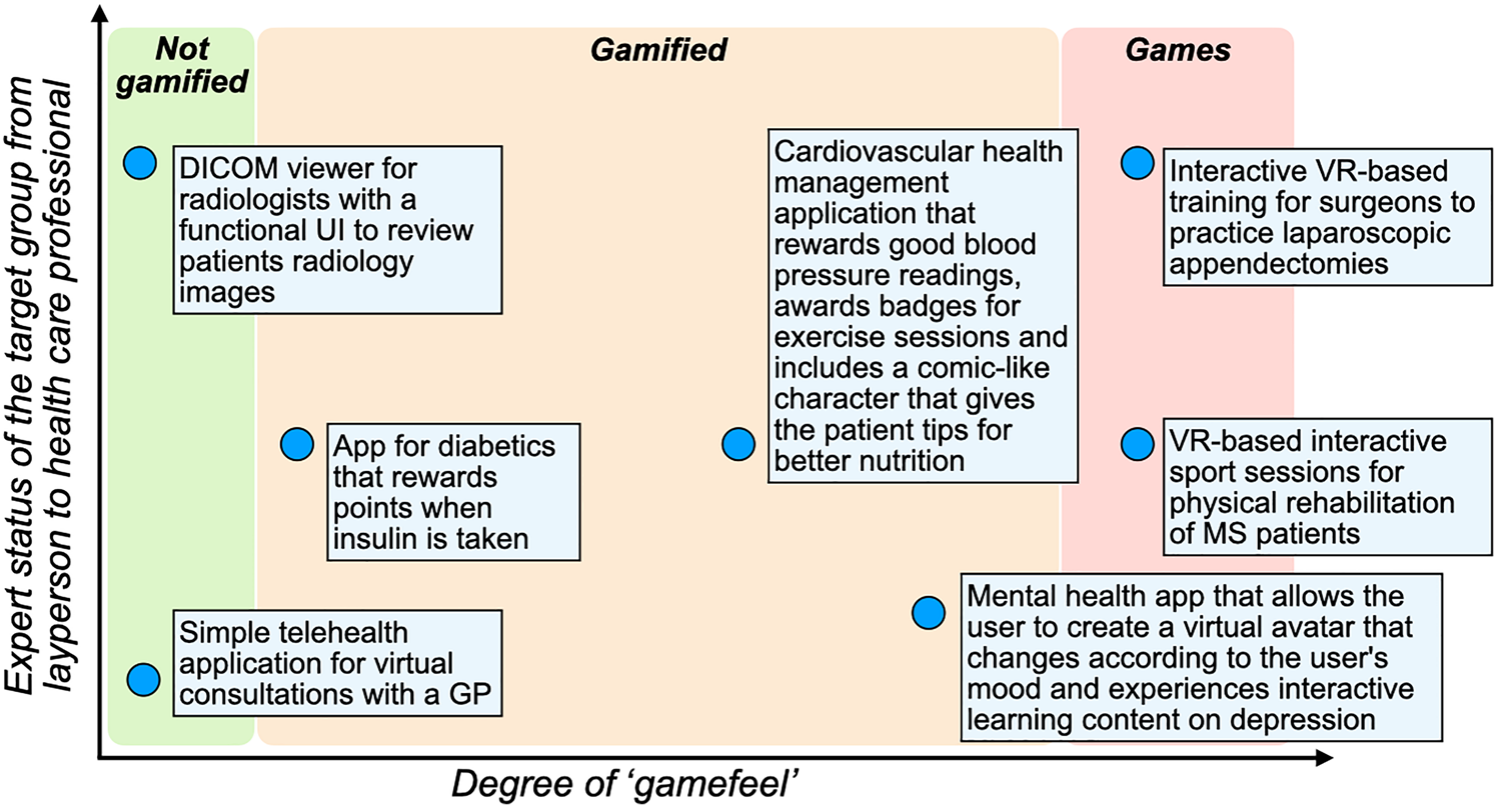
The user type and gamification space of mHealth apps. The range of current on-market apps is described according to their intensity of ‘gamefeel’ (x-axis) and their intended user group (y-axis). The degree of ‘gamefeel’ of the apps was based on the definitions used for this study^4,11,13,14^. The apps shown are based on the properties of on-market apps.

The integration of these game elements can be approached in two different ways. One is through the development of fully-fledged games, commonly referred to as ‘Serious Games’ (SG)^8^. Alternatively, specific game design elements (e.g., ‘points,’ ‘badges,’ or ‘leaderboards’) can be used within non-gaming apps, known as gamification^8–10^. The definition and distinction of both terms are still much debated. Various definitions exist, some of which are incongruent^7,10^. This is further complicated by the fact that gamification can be incorporated into apps to differing degrees, and in extreme cases, it is difficult to distinguish between gamification and SGs^11^. SGs are often defined as an “Interactive computer application, with or without significant hardware components, that has a challenging goal, is fun to play and engaging, incorporates some scoring mechanism, and supplies the user with skills, knowledge, or attitudes useful in reality […]^8^,” while gamification is often defined as “the use of game design elements in non-game contexts^9^.” This definition is limited^10,11^ and was extended by Sailer et al. in 2017, who defined gamification as the “[…] process of making activities in non-game contexts more game-like by using game design elements^4^.” SGs, however, are inspired by entertainment games in appearance and technology and often need specific hardware (e.g., powerful computers, VR headsets, or gaming controllers)^8,12^. In contrast, gamified apps utilize game elements like points, badges, high scores, leaderboards, and storytelling^4^ and generally have cheaper development and fewer hardware constraints than serious games. The differences in and application of these approaches are described in Fig. 1.

The number of gamified apps on the market is growing^15–17^, with many examples among fitness, business, and education apps^18,19^. A sector that is also growing is healthcare-specific gamified approaches^20,21^, which are increasingly linked to wearable devices (e.g., smartwatches) and are applied in chronic disease management, therapy, nutrition, and mental health^21–23^. A further grouping is apps as platforms for healthcare organizations such as insurance companies^20^.

The evidence on the effectiveness, safety, and benefits of gamification in apps is mixed. There is suggestive evidence of its beneficial effects on health-related behaviors in one systematic review, particularly in promoting physical activity, nutritional awareness, healthy eating, and medication adherence, with positive effects in 59% of included interventions and neutral or mixed effects in 41% of the included studies^22^, and a statistically significant but not clinical relevant improvement of knowledge (mean difference of 0.88 (0.05–1.75), 95% confidence interval, *p* < 0.05) and no effect on BMI in another meta-analysis^24^. Behavioral outcomes seem to be positively targeted, with some potential in mental health^22^. Yet, another systematic review indicated that gamification elements within mental health apps did not significantly boost the effectiveness of interventions or user adherence^25^. Gamification has shown a favorable influence on food selection and related nutritional behaviors in children and adolescents in the post-intervention phase (increase of the serving of fruits and vegetables of about 0.67)^24^. In diabetic populations, gamified interventions have been associated with improved medication adherence and HbA1c control (mean difference − 0.21; 95% confidence interval (− 0.37 to − 0.05); *p* = 0.01)^26^ and reduced high-fat food consumption in diabetes self-management^27^. However, the lasting effect of gamification is not yet fully understood, as shown by the unknown long-term effects on blood glucose control beyond a 12-month period^26^. In addition, insufficient technical implementation of gamification elements or the use of these elements without understanding the underlying psychological mechanisms limits user engagement^28^. A recurring limitation in serious games and gamification research is the lack of robust study designs and randomized controlled trials, making it difficult to draw concrete conclusions^22,24,27,29,30^.

When developing gamified applications for clinical practice rather than research purposes, new challenges might arise, such as ensuring clinical safety and managing risks associated with their use. Once gamified apps have a medical purpose, e.g., the treatment of specific diseases, they must comply with existing stringent regulatory requirements like all devices with a medical purpose. This includes obtaining clearance or approval as medical devices (MDs) and adhering to rigorous quality management frameworks encompassing development, release, audit, and clinical evidence of safety and efficacy. This procedure is not only intended to assess the potential benefits of an application, but primarily to mitigate the risks associated with the use of this product for end users, regardless of the actual clinical improvements. Globally, MDs, including gamified apps, fall under various regulatory jurisdictions, such as the EU’s Medical Device Regulation 2017/745 (MDR)^31^ and the US Food, Drug & Cosmetic Act (FFDCA)^32^. In the context of the EU, a device (e.g., software but also SGs) qualifies as a MD if, among other things, it is intended by the manufacturer to be used for “(…) diagnosis, prevention, monitoring, prediction, prognosis, treatment or alleviation of disease (…)”, injury or disability, or for the control or support of conception^31^. In addition to the binary classification as a medical device or not, many jurisdictions classify devices into certain classes (e.g., Class I to Class III) depending on the risk they pose to the user^31,32^. This risk classification is based on defined rules for different kinds of devices, e.g., software, surgical instruments, or invasive devices^31,33^. Supplemental Material 1 provides a detailed description of the steps for qualification as a medical device and for classification into specific risk classes with examples. Putting such a MD on the market without meeting these requirements, when identified by the regulator, would be followed by warning letters for removal of the product, followed by enforcement action, which could include heavy fines or even custodial sentences. The latter are rare, and regulators have limited capacity for market surveillance. Therefore, unapproved (and illegally on the market) apps persist in the app stores^34^. An investigation of drug-dosage calculators, which are also considered SaMDs, has shown that the regulatory compliance of these freely available apps is poor and that they pose a risk to patients^35^. Although there is no definitive evidence of widespread actual harm to patients caused by SaMD malfunctions^36,37^, the task of regulation is to prevent this as much as possible. Since most app-based MDs are distributed via the Apple and Google app stores, which are considered distributors and often importers under MDR^31^, they are responsible for ensuring conformity with regulations^38^.

Interpreting the regulatory clearance/approval process can already be challenging for non-software MD manufacturers or traditional software developers^39,40^. Interpretation is yet more challenging for cutting-edge technologies, including artificial intelligence (AI), generative artificial intelligence (e.g., large language models)^41–44^, or gamification. Here, both the interpretation of whether the intended purpose of the app qualifies as a medical device and their classification (grouping into regulatory ‘risk classes’ of medical devices) can be difficult. Also, since these apps could pose previously unknown risks, assessing their risks, including psychological effects, situation-specific interfaces, individualized user experiences, or device performance, can be challenging^45^.

Despite the increasing popularity of gamified health apps, it remains unclear whether, as a category, they are effective or meet the regulatory requirements placed on them to ensure public safety. Even though the regulatory path of gamified applications is the same as that of non-gamified applications, this relatively new technology poses particular regulatory challenges within existing legislations^45^. Existing regulations provide guidance on how to assess and mitigate the risks of certain aspects of SaMD, including audio-visual technologies^46^, the use of AI^47^, or cybersecurity^48^. However, gamification differs from these aspects covered by existing guidance due to the unique characteristics described above. Therefore, this study assesses the regulatory compliance of the EU’s most popular gamified health apps, available via the Google PlayStore and Apple AppStore. It examines the evidence supporting their medical claims (including diagnostic and therapeutic claims) and compliance with the EU’s MDR. The study includes a qualitative comparison of the regulatory compliance of the identified apps in the US. We hypothesize that (H1) many gamified apps are on the market without the required MD approval and that (H2) unapproved gamified apps on the market without the required MD approval have hazardous or uncontrolled aspects to their gamification that could lead to patient harm.

## Results

### General results

**Fig. 2:**
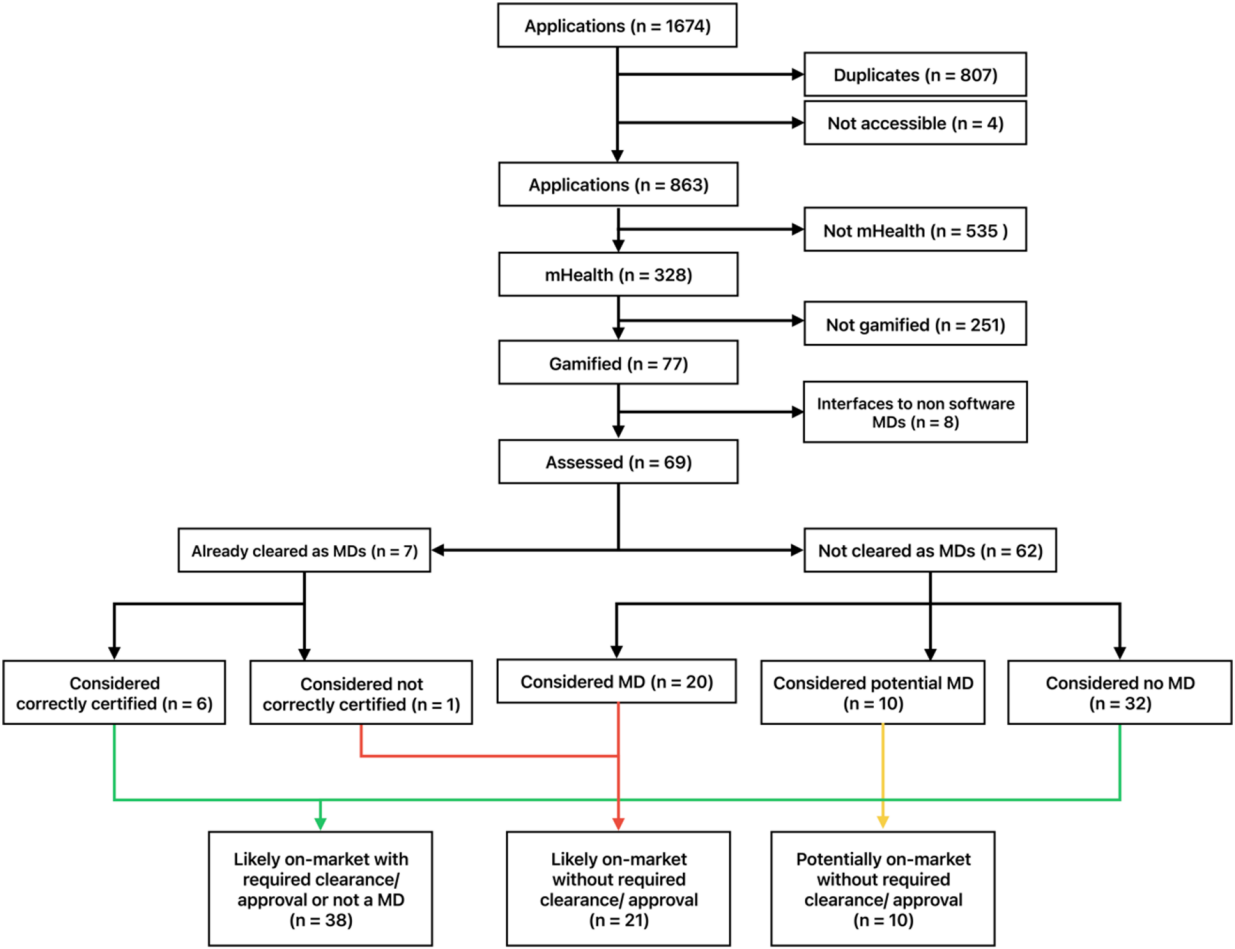
Selection process of apps. The included apps were in the Top 50 ‘free’ and ‘paid’ apps, as listed by the iOS AppStore and Google PlayStore, in the categories ‘Medical’ and ‘Health&Fitness’; and (ii) from the five EU Countries with the largest populations, Germany, France, Italy, Spain, and Poland, (these countries represent 66% of the total EU population).

The completed database contained 1674 apps, with the flowchart presented in Fig. 2. The search was conducted on the 27th of July, 2023. 807 apps were removed due to duplicates, and four could not be accessed since their sites were not retrievable in the app stores. The remaining 863 apps were then examined by the mHealth assessment panel. Of these, 328 met the criteria for mHealth apps. The gamification assessment panel examined those, and 77 apps were considered gamified. Eight apps were excluded since they functioned solely as interfaces for sensors or hardware medical devices. The remaining 69 apps were examined by the regulatory, clinical evidence, and risk expert panel. Seven of these 69 apps were already approved as medical devices in the EU, two according to the Medical Device Directive (MDD)^49^ and five according to MDR^31^. All five (of seven) MDR-cleared and one (of two) MDD-approved apps were considered to have the correct risk class and CE-marking under the regulations as they currently apply (allowing for the transition arrangements for the older MDD regulations). One MDD app was assessed as likely to be up-classified in its risk class under MDR (from MDD Class I to MDR Class IIa). All seven MD-approved apps were assessed as qualifying as MDs by the panel. Of the 62 not approved apps, 32 were considered ‘non-MDs,’ 10 were considered potential MDs, and 20 were considered MDs. The strength of agreement between the reviewers regarding the qualification as MD was *fair* (Fleiss' kappa = 0.40).

Apps that qualify as MDs have to follow a defined regulatory process. Considering all 69 assessed apps, 32 (46.4%) do not need to follow the regulatory process since they do not qualify as MDs. Six apps (8.7%) were rated by the panel as compliant with the required regulatory process. 10 apps (14.5%) were considered as potentially on the market without the required regulatory approvals, and 21 (30.4%) as on the market without the required regulatory approvals. The strength of agreement between the reviewers on regulatory compliance was *fair* (Fleiss' kappa = 0.37).

All seven approved apps and two not approved apps (13.0%) presented evidence in the form of peer-reviewed publications on their websites that demonstrate the effectiveness of their medical claims.

### Categorial analysis

**Fig. 3:**
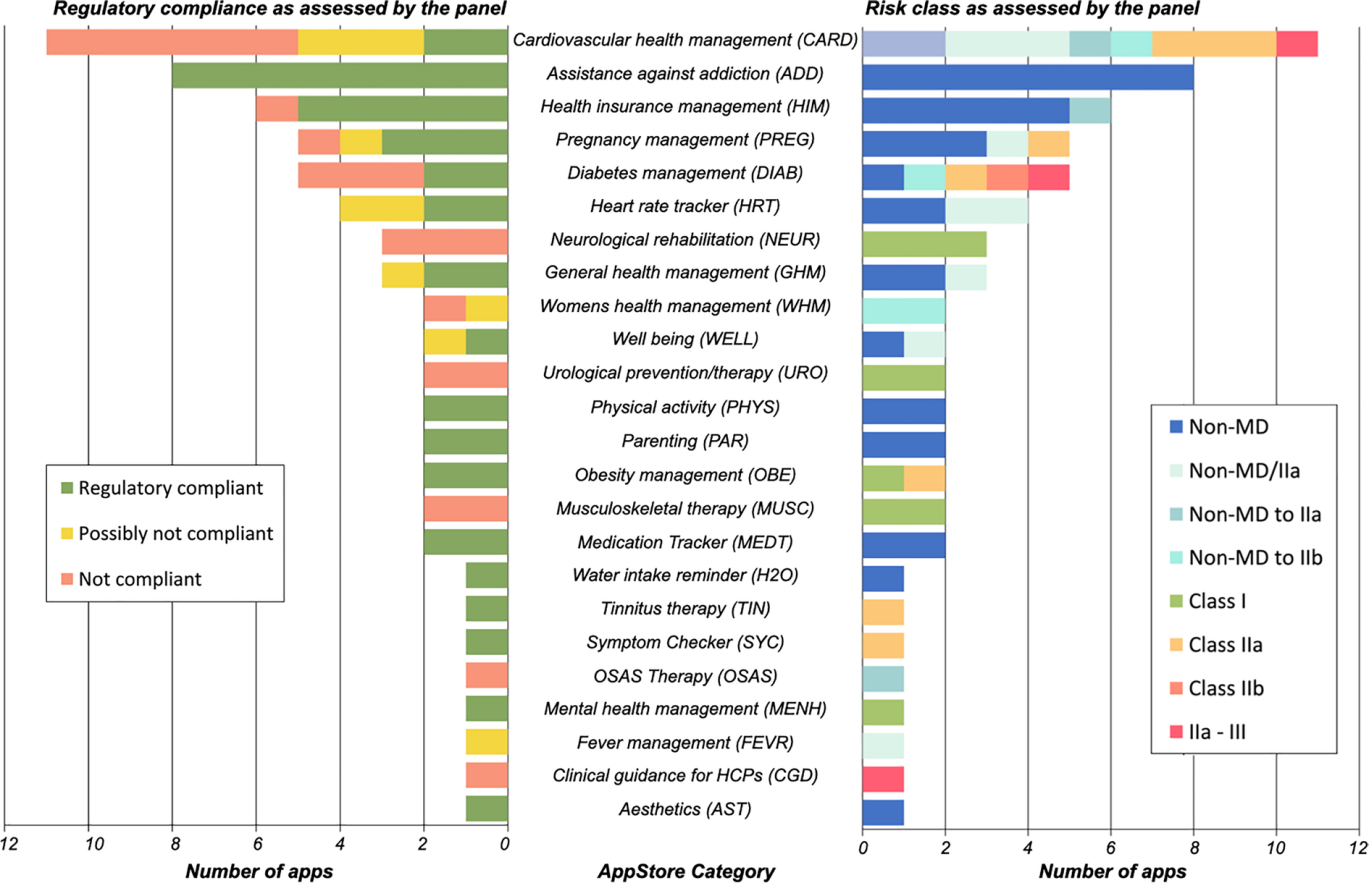
Regulatory assessment of apps by category. The left panel shows the regulatory compliance of the apps in each category. The right panel shows the assessed risk classes of the apps in each category.

The apps are distributed across 24 medical indication/intended purpose categories. The largest category of apps was *cardiovascular health management* apps (n = 11), followed by apps *assisting with addictions* (n = 8), *health insurance management* apps (n = 6), *diabetes management* apps (n = 5), and *pregnancy management* apps (n = 5). The remaining apps were distributed over categories, with low numbers per category (1–3 apps), belonging to categories including, e.g., *clinical guidance for HCP* apps or *symptom checker*. A detailed list of all categories can be found in Fig. 3.

The apps with the highest risk class as assessed by the regulatory panel can be found in the category of *clinical guidance for HCP* apps (Class IIa–III), *diabetes management* apps (Class IIb and Class IIb–III), and *cardiovascular health management* apps (Class IIa–III). All apps *assisting with addictions*, apps that foster *physical activity*, *medication tracker*, and *parenting* apps were considered ‘non-MD.’ The degree of compliance to applicable regulations varied greatly between app categories: in some categories, most or all apps were compliant with regulations (e.g., *symptom checker*, *mental health* apps), while in other categories, all or nearly all apps could be considered to not have the correct risk class and CE-marking under the regulations as they currently apply (*cardiovascular health management*, *neurological rehabilitation*, *diabetes management*, *urological prevention/therapy*, *musculoskeletal therapy*). Among the approved apps, two were intended for *obesity management* and two for *diabetes management*.

### Qualification as a medical device and risk classification

**Fig. 4:**
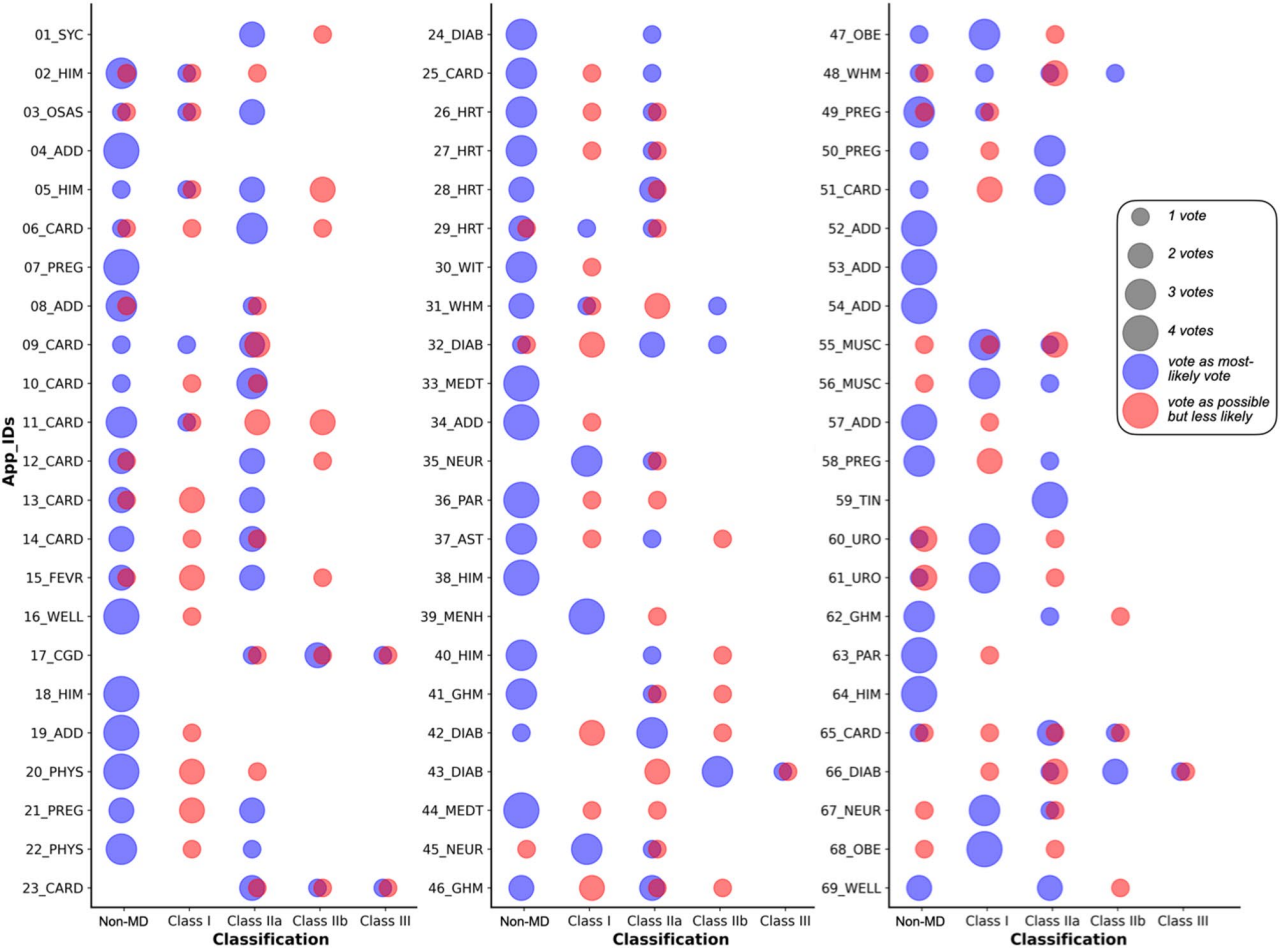
Distribution of assessed risk classifications for individual apps. The figure shows the distribution of the four ‘most-likely’ panel votes per app (blue) and the distribution of possibly but less likely votes (red) for the risk classification by the regulatory expert panel for the EU market. Each app has its medical indication/intended purpose category listed, as defined in Fig. 3.

32 (46.4%) apps were considered not to be classified as MDs under EU law, while 10 (14.5%) were considered potential MDs, and 27 (39.1%) were considered MDs. The reliability of agreement between the assigned ‘most-likely’ vote for risk classification of reviewers was *fair* (Fleiss' kappa = 0.33). In case of unanimous votes by the panel, ten apps would fall in Class I (14.5%), seven in Class IIa (10.1%), and one in Class IIb (1.4%). For non-unanimous votes, the range was quite broad. Four apps (5.8%) ranged between ‘non-MD’ and Class IIa, another three (4.3%) between Class IIa and Class III, four apps (5.8%) ranged between ‘non-MD’ and Class IIb and eight apps (11.6%) were considered ‘non-MD’ or Class IIa. A detailed presentation of the panel’s votes, the range of the panel’s ‘most-likely’ votes on the qualification/classification, and other qualification/classifications considered as possible but less likely by the panel are shown in Fig. 4.

### App store overview

21 (30.4%) apps were only available on Android, 27 (39.1%) only on iPhone, and 21 (30.4%) on both. The highest share of apps that might be on the market without required approval was on Android, with 14 out of 21 apps (66.7%) compared to 13 out of 27 apps (48.1%) on iPhone.

Most apps fell in the category of ‘Medical’ (n = 42, 60.9%). Of these apps, 27 (64.3%) could be considered MDs or potential MDs, but according to the expert panel, only 21 were on the market with correct risk class and CE-marking under the regulations as they currently apply. All seven already cleared/approved apps were in this category. Among the ‘Health & Fitness’ apps (n = 27, 39.1%), 10 (37.0%) were considered MDs or potential MDs by the regulatory panel. None of them had the required clearance/approval. 44 (63.8%) apps were free of charge, and 25 (36.2%) were in the ‘Paid’ section. Further details can be found in Table 1.

**Table 1:** Regulatory compliance of the mHealth apps in both major app stores, Apple AppStore and Google PlayStore.

Category	Total	Assessed as ‘non-MD’	Assessed as potential MD	Assessed as MD	Cleared/approved as MD	Likely on-market with required clearance/approval	Potentially on-market without required clearance/approval	Likely on-market without required clearance/approval
Android	21 (30.4%)	5 (23.8%)	4 (19.0%)	12 (57.1%)	2 (9.5%)	7 (33.3%)	4 (19.0%)	10 (47.6%)
iPhone	27 (39.1%)	14 (51.9%)	5 (18.5%)	8 (29.6%)	0 (0.0%)	14 (51.9%)	5 (18.5%)	8 (29.6%)
Both	21 (30.4%)	13 (61.9%)	1 (4.8%)	7 (33.3%)	5 (23.8%)	17 (81.0%)	1 (4.8%)	3 (14.3%)
Medical	42 (60.9%)	15 (35.7%)	4 (9.5%)	23 (54.8%)	7 (16.7%)	21 (50.0%)	4 (9.5%)	17 (40.5%)
Health& Fitness	27 (39.1%)	17 (63.0%)	6 (22.2%)	4 (14.8%)	0 (0.0%)	17 (63.0%)	6 (22.2%)	4 (14.8%)
Free	44 (63.8%)	21 (47.7%)	7 (15.9%)	16 (36.4%)	7 (15.9%)	27 (61.4%)	7 (15.9%)	10 (22.7%)
Paid	25 (36.2%)	11 (44.0%)	3 (12.0%)	11 (44.0%)	0 (0.0%)	11 (44.0%)	3 (12.0%)	11 (44.0%)

The ‘Total’ column indicates the count and percentage of apps out of the 69 assessed apps. Subsequent columns provide percentages based on the ‘Total’ count.

### Gamification analysis

The average degree of gamification, defined as the subjective perception of the effectiveness of gamification (‘gamefeel’) rather than a quantitative measure of the number of gamification elements, was assessed as medium (2.3 out of 5), with the highest degree of gamification assessed as 4. Five apps used the element ‘points,’ four the element ‘badges,’ six implemented ‘performance graphs,’ one included ‘avatars,’ and one included ‘storytelling elements.’ No app used ‘leaderboards’ or ‘multiplayer features’ to foster competition. Four apps were intended for cardiovascular health management, one for mental health management, one for diabetes management, one for pregnancy management, and one for neurological rehabilitation. Details can be found in Table 2.

**Table 2:** Characteristics of apps assessed in the gamification analysis.

App_ID	‘Most-likely’ risk class	Cleared/approved class	Points	Badges	Leader-boards	Performance Graphs	Avatars	Story-telling	Team-mates	Game-feel (1–5)
06_CARD	Class IIa			x		x				1.5
10_CARD	Class IIa		x			x				2
23_CARD	IIa–III		x	x		x				3
39_MENH	class I	MDR class I		x		x		x		2
43_DIAB	Class IIb	MDR class IIa + IIb	x	x		x	x			2.5
50_PREG	Class IIa		x							1.5
51_CARD	Class IIa					x				1
67_NEUR	Class I		x							4

Checkmarks indicate that the app included a certain gamification element. ‘Gamefeel’ is reported as the average value of all reviewer’s votes. Each app has its medical indication/intended purpose category listed, as defined in Fig. 3.

#### Analysis of app risks related to gamification

The numerically predominant risk identified across a subset of eight apps in the in-depth gamification analysis was the possibility of users being misled by incorrect medical information, predominantly attributed to software bugs, which could result from poor programming or inadequate testing, or attributed to conceptional problems in the design process, e.g., of interfaces. This risk was frequently classified as *moderate* in severity. The correlation between this risk and the gamification elements was generally assessed as *weak*, particularly in apps with a lower degree of ‘gamefeel’ (1 and 2 out of 5). Apps with a higher ‘gamefeel’ were assessed as having a stronger linkage between gamification and risk. These risks include the danger of users developing an over-reliance on the gamified tool caused by more compelling engagement mechanisms. Due to the assessment process based on ISO 14971, similar hazards, foreseeable sequences of events, hazardous situations, and harms in different apps could lead to different severities. The same applies to the association between gamification and risk, as this relationship depends on whether gamification elements alone have an impact on risk or whether other aspects of the app have an effect as well. Details can be found in Table 3.

**Table 3:** Specific risks of gamification elements in mHealth apps.

App_ID	Hazard	Foreseeable sequences of events	Hazardous situation	Harm	Severity	Association of gamification with risks
06_CARD						
*	Incorrect medical information provided to the user that misleads them about the severity, urgency, or treatment of a medical problem	Used by a user for the described medical use case and due to an underlying software error (possible cause: insufficient or unstructured testing by the manufacturer), incorrect blood pressure information is displayed to the user, along with incorrect findings/ analysis over time	The user chooses to act on the basis of incomplete information and either receives incorrect or delayed treatment or is at risk of iatrogenic harm in a medical setting	Deterioration of health	3	Weak
10_CARD						
*	Incorrect medical information provided to the user that misleads them about the severity, urgency, or treatment of a medical problem	Used by a user for the described medical use case and due to an underlying software error (possible cause: insufficient or unstructured testing by the manufacturer), incorrect blood pressure information is displayed to the user, along with incorrect findings/ analysis over time	The user chooses to act on the basis of incomplete information and either receives incorrect or delayed treatment or is at risk of iatrogenic harm in a medical setting	Deterioration of health	3	Weak
23_CARD						
**	Incomplete assistance due to limited app functions when in-app currency is no longer available	User engages with AI doctor, writes relevant health concerns using in app currency, needs to stop writing when he has no currency left, waits until next day to get his daily reward, continues chatting	The user chooses to act on the basis of incomplete information and either receives incorrect or delayed treatment	Deterioration of health, Delay of therapy	3	Strong
**	Incorrect medical information provided to the user that misleads them about the severity, urgency, or treatment of a medical problem	Misleading the user by providing contradictory information in the app regarding the context of its advice (i.e. advice labeled as coming from a specialized "AI" doctor who is clearly labeled as being able to provide specific advice on certain diseases and disease syndromes, contradicting a disclaimer stating that the app is intended for general health information). This misleads the user into believing that the app is a medical service or a reliable AI doctor. The user asks medical questions for which the underlying GPT-4 model, or prompt engineering provides false and dangerous medical information	The user chooses to act on the basis of the incomplete information and either receives incorrect or delayed treatment or is at risk of iatrogenic harm in a medical setting	Deterioration of health	3	Strong
*	Neglect of non-gamified health features of the app	The app has different main functions, such as step counting, blood pressure measurement, weight measurement, a chat function. (AI doctor) and calory counting. Some of those functions are highly gamified, e.g., the chat function. A focus on these can lead to a neglect of non-gamified features (e.g., blood pressure measurement) leading to an incorrect or delayed treatment	The user neglects relevant non-gamified health functions of the app leading to incorrect or delayed treatment	Deterioration of health	3	Strong
39_MENH						
*	Overreliance on the application	The user downloads the app, uses the daily mood tracking and typing features to assess their mental health, and begins to rely solely on the app for assessment. As a result, the user skips therapy sessions, believing that continued use of the gamified app is sufficient for their wellbeing	The User neglects an effective therapy in favor of the app and receives incorrect or delayed treatment	Deterioration of health, Delay of therapy	3	Moderate
*	Incorrect medical information provided to the user that misleads them about the severity, urgency, or treatment of a medical problem	The user suffers from a severe mental illness and relies too much on this digital application due to incorrect or inappropriate information	The user chooses to act on the basis of incomplete information and either receives incorrect or delayed treatment or is at risk of iatrogenic harm in a medical setting	Deterioration of health	3	Weak
43_DIAB						
*	Incorrect data entered by the user leads to incorrect calculations by the app and displayed values	The user enters data in the daily entry. Each entry is rewarded with points. 50 points are required to tame the "monster". The user can add made-up entries to gain more points. This can lead to incorrect information on carbohydrate intake, exercise and activities and thus to incorrect calculations of the inulin dosage	The user chooses to act on the basis of incomplete information and either receives incorrect or delayed treatment	Deterioration of health	3	Strong
*	Incorrect medical information provided to the user that misleads them about the severity, urgency, or treatment of a medical problem	Used by a user for the described medical use case and due to an underlying software error (possible cause: insufficient or unstructured testing by the manufacturer), incorrect blood sugar or diabetes information is displayed to the user, along with incorrect findings/analysis over time	The user chooses to act on the basis of incomplete information and either receives incorrect or delayed treatment	Deterioration of health	3	Moderate
*	Neglect of less gamified health features of the app	The app has different main functions, related to blood sugar management. Some of those functions are gamified by a challenge system. A focus on these can lead to a neglect of less-gamified features leading to an incorrect or delayed treatment	The user neglects relevant less gamified health functions of the app leading delayed treatment	Deterioration of health	3	Strong
50_PREG						
*	Incorrect medical information provided to the user that misleads them about the severity, urgency, or treatment of a medical problem	Used by a user for the described medical use case and due to an underlying software error (possible cause: insufficient or unstructured testing by the manufacturer), incorrect information on pregnancy is displayed to the user, along with incorrect findings over time	The user chooses to act on the basis of incomplete information and either receives incorrect or delayed treatment	Deterioration of health	2	Weak
51_CARD						
*	Incorrect medical information provided to the user that misleads them about the severity, urgency, or treatment of a medical problem	Used by a user for the described medical use case and due to an underlying software error (possible cause: insufficient or unstructured testing by the manufacturer), incorrect blood pressure information is displayed to the user, along with incorrect findings/ analysis over time	The user chooses to act on the basis of incomplete information and either receives incorrect or delayed treatment or is at risk of iatrogenic harm in a medical setting	Deterioration of health	3	Weak
67_NEUR						
*	Overreliance on application	The user interacts with the app, is very committed, neglects the lack of improvement due to the captivating gamified experience and seeks professional help later	The User neglects an effective therapy in favor of the app and receives incorrect or delayed treatment	Deterioration of health, Delay of therapy	2	Strong
*	The gamified approach does not accurately measure, monitor and treat visual acuity deficits, leading to user frustration and loss of willingness to engage in future approaches to improve visual acuity deficits	Used by a user for the described medical use case, and due to an underlying software error (possible cause: insufficient or unstructured testing by the manufacturer), the gamified approach does not accurately measure, monitor or treat visual acuity deficits, resulting in loss of engagement/adherence to future approaches to improve visual acuity deficits	The user is enthusiastic about the tool but over time realizes that the app is unsuitable/counter-productive, either through their own realization over time or through the advice of a healthcare professional. This leads to demotivation, frustration of the user and a loss of engagement/loyalty in future approaches to improve visual acuity deficits	Deterioration of health	2	Strong

The risk analysis was done according to ISO 14971^50^. The strength of the association of gamification elements with risks was reported as *none*, *weak*, *moderate*, or *strong*. *The risk is hypothetical, as it could occur with an app of this type, particularly if quality control is inadequate but has not been shown to have occurred with the analyzed apps—it is common to consider hypothetical/potential risks in risk assessments. **The risk is not hypothetical, as several tests have shown the app to provide incorrect or harmful advice.

### US comparison

Of the 69 apps assessed by the regulatory panel, 63 were available on the US market and were therefore also evaluated by a US regulatory expert. These included all seven apps with EU regulatory approval. According to the expert assessment, 11 of the 63 apps (17.5%) were considered as not qualifying as MD under US regulations, 41 apps (65.1%) would fall in Class I, 10 in Class II (15.9%), and zero in Class III. One app could not be assessed due to uncertainties of the intended use, an inadequate description, and insufficient material.

Compared with the EU regulatory assessment, more apps would qualify as MDs in the US (51 of 63, 81.0%) than in the EU (37 of 69, 53.6%). The proportion of apps assessed as being on the market without the required approval is smaller in the US (7 of 63, 11.1%) than in the EU (31 of 69, 44.9%). The expert attributed this to the fact that although the definition for Class I includes many functionalities/purposes in its scope, many of these products’ regulatory pathways would be through the self-declared enforcement discretion route^51^ or the self-declared 510(k) exempt route^52^ with a low accompanying regulatory effort for developers^53^.

**Fig. 5:**
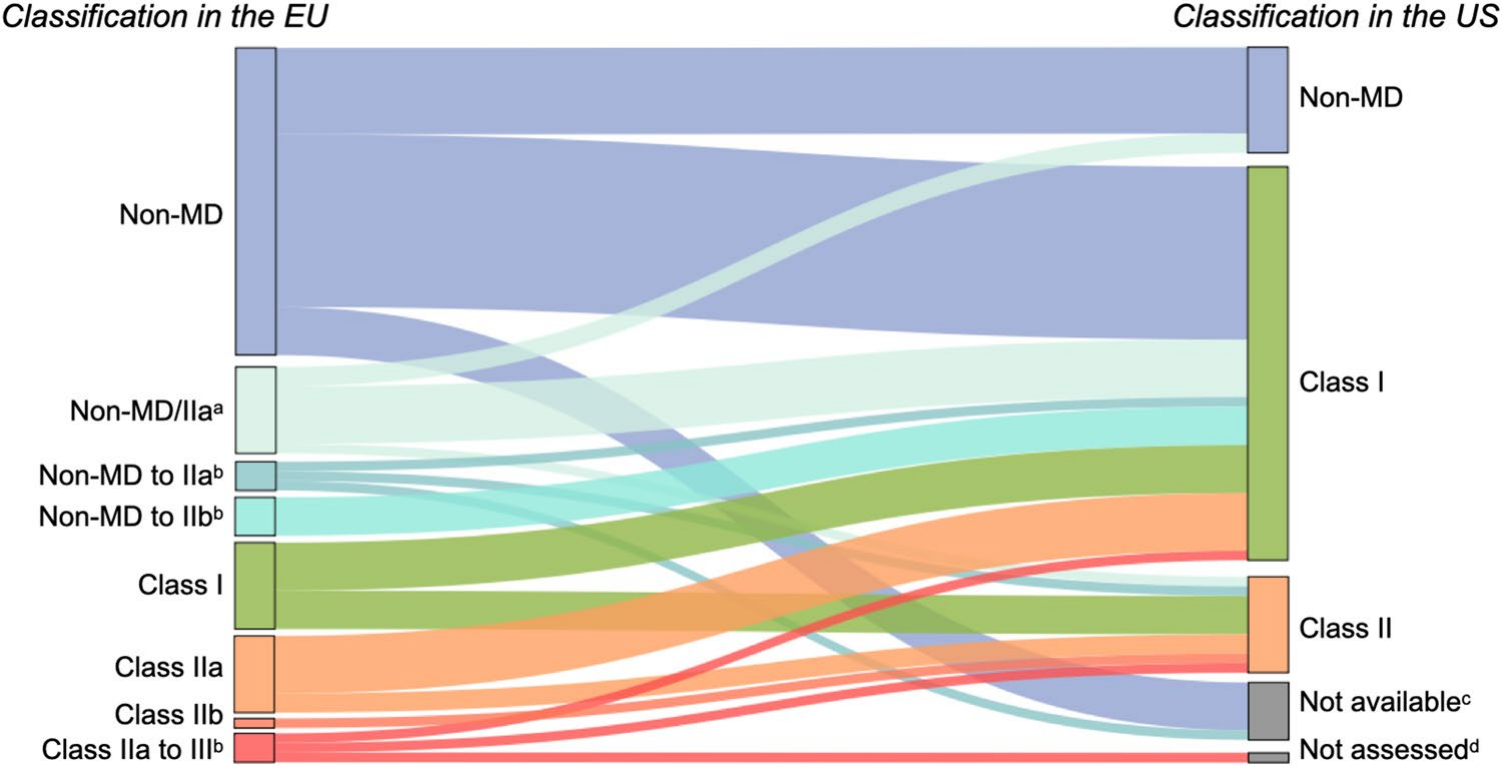
App risk classifications in the EU compared to the US. The Sankey diagram shows how groupings of EU-app risk class assessments map to US risk class assessments. All groupings of the EU-app risk class are shown on the left, considering tied panel decisions and the range of panel views. ^**a**^Tie vote with two votes for ‘non-MD’ and two votes for Class IIa. ^**b**^Tie vote with a distribution of votes among all specified risk classes. ^**c**^Four apps were not available on the US market and, therefore, not assessed for that market. ^**d**^The US regulatory status for one app could not be assessed due to uncertainties about its intended use.

Seven apps (11.1%) were considered on the market without the required regulatory clearance/approval, all of which would fall in Class II in the US. One of these apps has already been approved according to MDD in the EU. Its available evidence would, however, not be sufficient for the FDA approval process. In contrast to the EU, the three apps for neurological rehabilitation fall into Class II in the US (these were judged to be in Class I in the EU). The risk of a cardiovascular health management app that uses LLMs was assessed as lower in the US (Class II, EU: Class IIb or III). Details can be found in Fig. 5.

## Discussion

### Principal results

Our study has several findings that have implications for regulatory oversight and market compliance of publicly available gamified mHealth apps in the EU. Out of 863 apps analyzed, 69 were gamified mHealth apps. The panel considered 37 apps (53.6%) as MDs or potential MDs, necessitating appropriate clearance. Only 7 (10.1%) of these apps were already considered to have the CE-marking under the regulations as they currently apply, 6 of them in the appropriate risk class. One app was judged to face an up-classification with the transition from MDD to MDR on 31 December 2028^54^. In total, 31 (44.9%) of apps were assessed as not or potentially not compliant with the regulatory requirements, which confirms our first hypothesis (H1).

Additionally, only 9 (13.0%) of the apps provide evidence for their effectiveness; among them, all seven cleared/approved apps^4,22,24,27,29,30,45,55,56^. There is therefore a large gap in evidence for the largest segment of gamified apps and serious games. It is concerning that there is lack of clinical evidence (or the non-publication of this by the manufacturers) and a lack of appropriate regulatory approval, as lay people with no medical training could download these consumer-facing apps.

Only 14.5% (n = 10) of the assessed apps would fall into a low-risk category (Class I under MDR), whereas 39.1% (n = 27) could be classified as Class IIa or higher*.* MDs that fall into Class I, based on the MDR rules, have an easier pathway to regulatory approval since they can self-declare their conformity with the MDR without the involvement of a notified body^31,57^. In comparison, higher-risk apps must be developed within a sophisticated and certified quality management system, bringing many quality requirements and increased regulatory burden to market access and involving a notified body for the conformity assessment^31,53^. This process is presented in detail in Supplemental Material 1. It is highly unlikely that any of the 23 (33.3%) apps that are on the market without a CE-mark but assessed as Class IIa or higher will have undergone any standardized process of design, testing, risk assessment, and post-market surveillance, which are judged necessary by legislatures for apps in these categories.

The reliability of agreement among reviewers on this binary decision (MD or not MD) was *fair* (Fleiss kappa = 0.40), while the reliability of agreement for the risk classification itself (non-MD to Class III) was noticeably lower but still *fair* (Fleiss’ kappa = 0.33). This could be due to different reasons: firstly, a large variation of the reviewer’s perspectives indicating the challenge, even for experts, to classify gamified apps; secondly, a regulatory framework that is ambiguous; and thirdly, a regulatory framework challenged by the continuous emergence of new software approaches and app types that were not anticipated when the regulations were written. We suspect that the reason for this lies in a combination of all three explanations, considering existing evidence that the practical classification of MDs is ambiguous and discussed by courts^58^ and among experts^59^. Additionally, the ruleset for SaMD remains unclear and relatively broad, as stated in MDCG 2019–11^60^. Some experts have even challenged whether any mHealth apps can be classified as MDR Class I devices, and some competent authorities have taken this view, refusing to accept the registration of any Class I apps^61^, while other experts and competent authorities have accepted the registration of Class I apps. This ambiguity is highlighted by the classification assessments of individual apps in this study, often ranging between Class I and IIa (Fig. 4).

### Specific risks linked to gamification

Our assessment shows that gamification was used in both approved MDs and in apps with a clear medical purpose that are available through the app stores despite lacking the required clearances/approvals. The second research question centered on whether the observed lack of compliance means risks for patients that might be related to gamification. The presence or absence of gamified elements does not solely determine an app’s qualification as a medical device since, here, the app’s intended purpose, which is defined by the developer, is crucial. However, gamification elements are of great importance for the risk assessment process, an integral part of the MD approval process, particularly when they are integral to the app’s medical purpose. Thus, they may affect the MD’s risk classification. While it is likely that apps that are approved/released as MD have considered the risks associated with gamification in their design, risk assessment, usability engineering, and mitigation measures as required by the regulations, it is highly unlikely that any of these formalized approaches to safe design have been followed for apps that are on the market without the required clearance/approval.

The in-depth analysis of a subset of eight apps was focused on specific risks associated with gamification elements and harms that could emerge from the failure of such elements. In a standardized approach defined in ISO 14971^50^, we have described risks related to hypothetical scenarios as well as risks that occurred while testing the app. This approach would be a requirement for the approval of MDs.

Our results show that for most of the apps examined, the scenarios of failure of gamification elements posed little risk to patients. This is due to the following: (i) the main risk to users was being misled by incorrect medical information, resulting in mild to moderate harm; and (ii) most of the apps studied had a low level of gamification, and we assessed that there was a low association between gamification and risk in the app. Some of these risks could be caused by software bugs, which could be addressed through updates, while others may stem from foundational design flaws requiring more substantive revisions. For apps with a higher degree of gamification, we found a stronger link between risks and gamification elements.

Of the eight apps included in the in-depth analysis, three had a strong connection between gamification and risk and possessed potentially hazardous or uncontrolled aspects to their gamification that could lead to patient harm. The first app (23_CARD) provided an interactive chat tool powered by ChatGPT-4 (a large language model-based chatbot^62^), branded as an “AI doctor.” This tool claims to offer health advice based on deep learning algorithms, presenting a user-friendly interface adorned with comic-style 3D elements and avatars. Its gamification strategy hinges on a credit-based reward system designed to encourage daily logins and advertisement viewing, using gift timers and push notifications to foster habit-forming user behaviors. The number of interactions with the chatbots is limited by an in-app currency, which could lead to incomplete health assistance. The user would then rely on partial information, potentially resulting in delayed or incorrect medical treatments. Moreover, the app risks disseminating incorrect medical information, which occurred during the testing, misleading users about the severity or treatment of their conditions by providing contradictory advice under the guise of a specialized “AI doctor.” This misinformation could inadvertently direct users to make harmful medical decisions based on incomplete or inaccurate data. Additionally, since some of the app’s features are non-gamified while others are, the app poses the risk that patients neglect these non-gamified features (e.g., blood pressure management), leading to incorrect or delayed treatment.

The second app (43_DIAB) for use by diabetics incentivized users to enter their blood glucose measurement data by awarding them with ‘points.’ It used this data to calculate self-application dosage amounts of insulin. The app in question uses only five out of 81 items in the questionnaire for insulin dosage calculation and also rewards entering ‘0’ as a value (e.g., if no insulin was taken). If those constraints are implemented in a poor way (e.g., no option for adding ‘0’ as a value and still getting rewarded), or no consistency check of the input is performed, the user could ‘cheat’ to gain more points, e.g., by adding impossible hours of training or carbon intake, wrong insulin injection dosages. Those wrong values could potentially lead to false analyses and health recommendations by the app and hence might lead to lower clinical outcomes or, in a worst-case scenario, to high-risk situations for the patient, e.g., in hypoglycemia, if too much insulin is injected. An additional risk identified is that some features of the app are less gamified than others. This could lead to a neglect of the less gamified features, leading to incorrect or delayed treatment. We acknowledge that we have not had the opportunity to review the material provided for the regulatory process for this specific app. These reports would include both usability and clinical testing and may demonstrate that the approach taken in the app is indeed safe.

The third app (67_NEUR) was rated as having a high ‘gamefeel’, although it only has one gamification element as defined by Sailer et al. (2017). Even if it cannot be described as a fully immersive, full-fledged game due to its limited visual presentation and the absence of relevant game aspects, it still represents an edge case. The gamified character in this app was much more strongly linked to the intended purpose than in the other applications analyzed, leading to a limited immersive experience for the users. As a result, the connection between the gamification elements and potential hazards was higher than in apps that included gamification elements but no immersive experience. One gamification-related risk identified in this and several other apps was that of over-reliance on the apps due to their engaging design and the stimulation effect of gamification. In scenarios of app malfunction, gamification of this nature could exacerbate delays to the start of effective treatment, as the gamification could promote ongoing use. The stronger the gamification was linked to the intended purpose of the app, the stronger the connection between risk and gamification was among the apps analyzed. Whilst no fully immersive SGs could be included in our study, 67_NEUR could point to interesting future research. Since the intended medical purpose of immersive SGs is particularly strongly related to gameplay, the connection between the degree of gamification and risk observed in our study could be investigated better.

Considering the complex and varied link between gamification and risks, Hypothesis 2 could neither be definitively accepted nor rejected. However, a small number of dangerous gamified apps were identified on the market that are not approved/cleared and where there is a clear need for action by the regulatory authorities and the app store in question (Google PlayStore).

### Implications for the app stores

Since Google and Apple have an effective duopoly on the mobile market^63^, they play a pivotal role in distributing consumer-facing health apps. As importers or distributors under MDR^31^, the app stores are legally obliged to ensure regulatory compliance of the apps they offer^38^. Our results on gamified apps support previous findings on other types of mHealth apps that neither Apple nor Google adequately meet those requirements^34,35^. This study shows that the availability of unapproved apps, despite the legal requirement for MD approval, is greater on the Google PlayStore (42.9%) than on Apple’s AppStore (35.4%). This observation is even more pronounced for gamified apps exclusive to the Google PlayStore, where 66.7% are unapproved despite the assessment in this study as requiring l MD approval.

Additionally, the naming convention of the categories in both app stores remains unclear. Most apps considered in this study as MDs are in the ‘Medical’ category (n = 27), but some are also found in the ‘Health&Fitness’ category (n = 10), and in this latter category, none of which are adequately cleared/approved. While it was expected that a lot of MDs would be found in the ‘Medical’ category, the high number of MDs in the ‘Health&Fitness’ category was surprising since fitness apps are generally not considered MDs^31,33,60,64^. A clear categorization could make distinguishing between MD and non-MD apps easier for users and HCPs.

Furthermore, apps communicate their MD status and associated risks in various ways. Some explicitly mention their regulatory classification and potential risks in their descriptions or supplementary documentation, such as websites or publications, and some do not at all. This inconsistency makes it difficult for users and HCPs to assess an app’s regulatory status and associated risk.

A balanced approach is necessary for app stores to address those challenges. Although regulatory bodies have the responsibility of approving new MDs, their limited resources make it difficult to provide complete oversight of all newly published applications. Here, the role of the app stores as market entry points becomes critical. Since they already have expertise in the automated screening and analysis of applications, they could assure regulatory approval or certification for apps with a medical purpose and implement basic checks to ensure content accuracy^34^. The aim of that is to make it impossible to download clearly unsafe and illegal products from the app stores.

### Assessment of the US approval status

Comparing the EU market with the US, we found a higher proportion of MD apps in the US. Nonetheless, due to the FDA’s enforcement discretion rules, fewer apps were marketed without the required approval in the US compared to the EU. This goes hand-in-hand with our finding that most assessed apps would fall into Class I and, therefore, be under enforcement discretion. The observation of a more flexible US regulatory classification approach for low-risk apps is consistent with the findings of other studies^53^. We observed that the FDA product classification database^65^ provided greater transparency around regulatory decisions and evidence than the EU counterpart database, which is only partially operational and will provide less transparency on completion^66^.

This poses two main implications for the EU. Firstly, the US system enables low-risk Class I digital apps to reach the market more rapidly, potentially spurring faster innovation^53^. Secondly, the easy access to information on regulatory decisions and evidence from the FDA databases provides openness on app classification decisions and associated evidence, which is lacking in the EU and is not planned for in the EU regulatory framework^31^. The greater US openness allows all stakeholders, including clinicians and the public, to examine the basis for regulatory approvals.

### Implications for the regulation of gamified MDs

Our analysis shows that many gamified mHealth apps would be considered as MDs under current regulations. As such, gamified MDs, like non-gamified MDs, must comply with the applicable laws regulating them. While the general compliance of mHealth apps (gamified and non-gamified) was questioned by other researchers^35^, the compliance issues might be caused by different reasons, including unawareness or insufficient guidance documents, although existing regulations provide guidance on certain aspects of SaMD, including audio-visual design^46^, AI^44^ and cybersecurity^48,67^. For gamified apps, this lack of compliance could be caused by a notable gap of guidance documents addressing the intricate relationship between gamification elements, engagement design, and their impacts on health outcomes. The unique characteristics of such apps should be considered in the current regulatory approval process along with other aspects of the app’s safety and performance within the assessment, validation, and regulatory frameworks. A tailored framework, guidance, or standard should delineate the specific risk categories associated with gamification and outline the required steps for design and human factors evaluation, as well as for clinical evaluation, considering the prolonged and dynamic user activity characteristics of gamified apps. Such a framework should complement existing regulatory legislation, but not replace them. They are intended to provide guidance on how these products are manufactured and how their risks can be assessed within the framework of the applicable laws. Similar guidance documents exist for other risk aspects of MDs, e.g. cybersecurity^48,67^ or AI^47^. These horizontal features are present in many MDs and create additional risks, while the corresponding guidance documents help to assess and mitigate them. Our paper is a step towards how this can also be done for gamification. The increasing number of gamified health apps and the ambiguities around current regulations presented in this study underline the necessity for specific guidelines.

Additionally, there is a need for a more transparent and accessible system to build trust in the EU medical device landscape, as has been recognized for implantable medical devices in the past^68^. This particularly applies to gamified mHealth apps, of which, in the judgment of the expert panel in this study, a surprisingly high proportion are on the market without the required approvals.

### Limitations

This analysis has several limitations. The inclusion criteria limit assessed apps to the most populous EU countries, and this potentially limits the generalizability of results to all EU member states. Additionally, due to feasibility reasons, we had to limit our search of the app stores to the categories ‘Medical’ and ‘Health&Fitness’. Thus, we might have missed a small number of apps that have been misclassified by the app stores or developers. Assessment panel members had free choice of whether to download, install, and explore the functionality of the mHealth app or to assess the app based on the developer-provided descriptions, images, and videos (on the app stores, developer websites, and research publications). A minority of apps were downloaded. This is, however, compatible with the assessment of apps based on the developer-stated claims as they relate to the developer-stated functionality, which is also the core approach of the regulatory approval process. Only mobile apps were included in this study.

The definition of gamification and of gamification elements is still an area of discussion among researchers; an agreed consensus definition does not yet exist, and multiple researchers propose different lists of gamification elements^4,22,69,70^. We used the definition proposed by Sailer et al. (2017)^4^, which is widely recognized. However, competing definitions also exist^9,11,13^, which could limit our findings.

The identification of evidence on existing apps could be limited as developers of apps, whether MD or non-MD, are not obliged to put evidence in the public domain. As the EU EUDAMED database is still under development, there is no single source to definitively search a list of approved MDs and their risk class. A detailed search of the app store descriptions, the developer’s websites, research publications, and a general internet search was conducted. The qualification of apps as MDs in the EU is largely based on the manufacturer’s reported intended purpose. It was not always possible for the assessment panel to base their decisions on a stated intended purpose since not all apps clearly stated this. In these cases, the panel’s judgment was based on all available information on claims and functionality.

The analysis of specific risks of gamification was only conducted for a subset of eight apps, which had to be available in German and English, free of charge, available in the Google PlayStore, and accessible without a doctor’s prescription. Additionally, the degree of gamification was judged on a self-developed arbitrary scale. Thus, findings about the intersection of risks and gamification could be limited by the arbitrary character of this scale.

## Conclusion

In the emerging field of gamified health apps, few commercial products meet the strict standards of medical device regulations, in particular, the EU’s MDR. Potentially unsafe and illegal products should not be available in the app stores, and the stores themselves and regulators should take action to remove them from the market in accordance with the applicable laws regulating MDs. Therefore, app stores must implement and enforce appropriate review procedures so that healthcare professionals and consumers can have confidence in the safety and efficacy of the apps they download. While the level of compliance for such apps is inadequate, the specific level of risk for most apps remains uncertain. In many cases, we found that there is only a weak link between the gamification elements and the potential risks of the apps. However, for the apps identified that have a high degree of gamification, the link between risks and gamification elements was stronger. This ambiguity highlights the need for further research to better understand and address the unique challenges that gamified health apps present in regulatory and safety contexts.

## Methods

### Overview

We used a 4-step approach adapted from other studies^14,71,72^. First, a database of apps was set up and cleaned. Second, all apps were assessed against defined criteria to determine whether they could be considered mHealth apps. Third, the remaining apps were evaluated to see whether they could be considered gamified. Fourth, the remaining apps were considered by a panel of regulatory experts who assessed their regulatory compliance. Each assessment was carried out using defined evaluation criteria. To assess whether the hypothesized lack of compliance, if confirmed, means risks for patients that might be related to gamification, an in-depth analysis of the association between gamification and risks of a subset of apps following an approach defined in ISO 14,971 was conducted. Additionally, a qualitative comparison of regulatory compliance in the US was conducted for those apps on the US market.

The statistical analysis was conducted using Python version 3.11.6 and the following libraries: pandas, NumPy, and statsmodels. Data visualizations were created using Matplotlib. To calculate the reliability of agreement between the regulatory panel members, a Fleiss’ kappa measurement was performed^73^; the interpretation was based on Landis and Koch^74^.

### Database setup

Since the app stores are organized regionally, and no EU-wide top list exists, each country had to be assessed individually. To keep the total number of apps manageable, only apps from the five EU countries with the largest populations, Germany, France, Italy, Spain, and Poland, which comprise 66% of the EU population, were included^75^. Besides apps listed in the Category “Medical” in both app stores, we also included apps listed in the heterogeneous category of “Health and Fitness,” which includes apps with a medical purpose, e.g., cardiovascular health tracking apps and non-health related apps like hiking navigation apps. The Top 50 “free” and “paid” apps as listed by the stores in the categories “Medical” and “Health and Fitness” in the iOS AppStore and Google PlayStore in the selected countries were identified through the search function of the web versions of the iOS AppStore and the Google PlayStore on the 27th of July, 2023 and included in the study database. The manufacturer’s name, store link, rank, and operating system were listed for each app. The database was set up using Microsoft Excel for Mac Version 16.78.

App store listings were systematically reviewed, and duplicates, identified by the name of the app, store URL, and developer identity, were removed. Multilingual duplicates were omitted. Complimentary free versions of premium apps, inaccessible apps, and collections of multiple apps were also excluded.

### Materials for the regulatory expert assessment

For all gamified mHealth apps, a store link and the main features of each app were provided. Since there is no standardized way in the app stores to report the state of regulatory approval and risks of an application, for each app, the EUDAMED database, the FDA databases, the DiGA directory (a directory of Digital Health Applications that can be prescribed by physicians and psychotherapists and are reimbursed by health insurers)^76^, the manufacturer’s website, and the app were searched for existing clearance/approval. If the app had already been cleared/approved by any regulatory body, all linked and publicly available materials were presented to the panel.

Evidence for the apps was searched in a systematic literature review via Google Scholar and PubMed using the search terms “[app name]” and via an internet search engine search with Google using the search terms (“[app name]” AND study). Additionally, the websites of the manufacturer and, in case of existing clearance/approval, the according database^65,66,76^ were screened. All identified peer-reviewed articles were presented to the panel members.

### Selection and assessment of apps

#### Composition and expertise of the panels

Three different panels were involved in the assessment stage of the applications. The first panel for the mHealth assessment consisted of one physician and game developer (author O.F.), one public health and gamification researcher (author K.J.W), and one expert in medical device software (author Q.S.).

The second panel, responsible for the gamification assessment, consisted of one physician and game developer (author O.F.), one public health and gamification researcher (author K.J.W), and one developer of serious games (author M.H.).

The third panel, responsible for the regulatory assessment, consisted of one professor for regulatory science and consultant for regulatory affairs (author S.G.), one professor of medical device regulatory affairs and a former senior medical officer at the Health Products Regulatory Authority (HPRA) (author T.M.), one digital health consultant (Author P.W.), and Regulatory Consultant and former Senior Technical Assessor at the UK Competent Authority (MHRA) (Author A.S.P.).

#### mHealth assessment

The cleaned dataset was examined by three reviewers who independently assessed in a binary decision whether an app could be considered a mHealth app based on predefined criteria applied to the descriptions and available picture and video material in the app stores and on the manufacturer’s website. The reviewers used the definition of the WHO extended by Tomlinson et al. to determine whether an app is a mHealth app. Those were defined as “(…) medical and public health practice delivered on digital devices, covering the use of mobile phones to improve point of service data collection, care delivery, and patient communication to the use of alternative wireless devices for real-time medication monitoring and adherence support^77,78^.” All apps that did not meet this definition were excluded. Each app was categorized according to its primary purpose, as stated in its description. Regarding non-English descriptions, two translation tools, “Google Translate” and “DeepL,” were used, and the resultant translations were checked for consistency. For German and French apps, the translation quality was verified by a native speaker. All apps from Spain, Italy, and Poland provided versions of the app and the app store page in English, which served as a base for the consistency check. In the case of divergent opinions between the reviewers, this was recorded, and the assessment of the majority of the reviewers was followed.

#### Gamification assessment

The dataset of the remaining apps was examined by three reviewers who independently assessed in a binary decision whether an app could be considered gamified based on predefined criteria applied to the descriptions and available picture and video material in the app stores and on the manufacturer’s website. The reviewers used the definition by Sailer et al., 2017^4^. The authors describe gamification as making activities in non-game contexts more game-like using the following game design elements. “Points” are awarded for specific accomplishments in a game. They signify a player’s progress with experience, redeemability, or reputation points. “Badges” are visual symbols of player achievements. They validate a player’s successes, serve as status symbols, provide feedback, and can influence player decisions. “Leaderboards” rank players based on their achievements compared to others. “Performance Graphs” are visual representations comparing a player’s performance to past results. “Meaningful Stories” are narratives that contextualize in-game activities, adding depth beyond scoring points. “Avatars” are visual icons representing players in the game. Players often choose or design them. “Teammates” are other players or computer-controlled characters promoting dynamics like conflict, competition, or cooperation^4^. This list by Sailer et al., however, is not necessarily exhaustive, with multiple competing lists existing^22,69,70^. Any apps that did not meet the definition were excluded. Additionally, apps that function primarily as input software for a specific other device (e.g., a sensor) were excluded as they do not function in isolation to deliver a medical purpose.

#### Regulatory expert assessment

A panel of four experts in medical device clinical evidence, risks, and regulation examined all remaining apps. Each reviewer was provided with the complete list of 69 apps in randomized order. The assessment was performed individually without communication between assessors.

The assessment of the regulatory panel was based on the given definition of MDs in the Regulation (EU) 2017/745 of the European Parliament and of the Council of 5 April 2017 on medical devices^31^. Here, a MD is defined as “any instrument, apparatus, appliance, software, implant, reagent, material or other article intended by the manufacturer to be used, alone or in combination, for human beings for one or more of the following specific medical purposes: diagnosis, prevention, monitoring, prediction, prognosis, treatment or alleviation of disease; diagnosis, monitoring, treatment, alleviation of, or compensation for, an injury or disability; investigation, replacement or modification of the anatomy or of a physiological or pathological process or state; providing information by means of in vitro examination of specimens derived from the human body, including organ, blood and tissue donations, and which does not achieve its principal intended action by pharmacological, immunological or metabolic means, in or on the human body, but which may be assisted in its function by such means^31^”. The risk classification was based on ‘Rule 11’ of the MDR, which states that “Software intended to provide information which is used to take decisions with diagnosis or therapeutic purposes is classified as class IIa, except if such decisions have an impact that may cause: death or an irreversible deterioration of a person’s state of health, in which case it is in class III; or a serious deterioration of a person’s state of health or a surgical intervention, in which case it is classified as class IIb. Software intended to monitor physiological processes is classified as class IIa, except if it is intended for monitoring of vital physiological parameters, where the nature of variations of those parameters is such that it could result in immediate danger to the patient, in which case it is classified as class IIb. All other software is classified as class I^31^,” on the MDCG 2021–24 Guidance on classification of medical devices^33^ and on the expert’s interpretation and knowledge of the application of those rules. A standardized scale was used for the assessment based on the regulatory legislation. All experts used those documents in the past and were familiar with their content and rules. Additionally, a set of risk class definitions based on those guidelines was provided to the panelists to establish a common ground and understanding of the applicable rules. An application could either be assessed as “Non-MD” if it does not qualify as a MD in the eyes of the expert, or it could fall in one of the four risk classes defined by the regulatory legislation, “MD Class I,” “MD Class IIa,” “MD Class IIb,” or “MD Class III.” Criteria used by the panel for the risk classification are provided in Table 4, and example apps for each risk class are shown in Supplemental Material 1.

**Table 4:** Risk classification framework.

Risk class	Definition
Non-MD	Fulfills none of MDR definition of medical device
Class I	Includes (for those countries/states that ‘allow’ Class I software) non-therapeutic and non-diagnostic applications and non-monitoring (monitoring interpreted as recording a medical signal/measurement over time explicitly for the medical tracking of disease progress . It is not interpreted as collection of any medical information, e.g. number of completed exercises), including disease management applications, were e.g. a specific program of physical exercises are not considered explicitly therapy
Class IIa	Includes software intended to provide information which is used to take decisions with diagnosis or therapeutic purposes (without a risk of death or serious harm) and software intended to monitor non-vital physiological processes
Class IIb	Includes software intended to provide information which is used to take decisions with diagnosis or therapeutic purposes (with a risk of serious harm) and software intended to monitor vital physiological processes, where the nature of variations of those parameters is such that it could result in immediate danger to the patient
Class III	Includes software intended to provide information which is used to take decisions with diagnosis or therapeutic purposes (with a risk of death / irreversible harm)

This framework was based on the rules provided in the MDR^31^ and on additional guideline documents^33,64,79^. All panelists had access to this framework and applied it in their assessment.

Every reviewer had to: (i) report the classification into which the app is likely to fall, with an assignment of the “most-likely” classification and any number of less likely but also possible classifications; (ii) report their degree of confidence in their “most-likely” classification assignment, on a scale from 1 (low confidence) to 5 (high confidence); and (iii) if an app had already regulatory clearance/approval, decide whether the app could be considered as adequately cleared/approved (particularly important as lower risk classes are self-declared by developers ). The judgment was based on the intended purpose, as presented by the developer. Assessors were instructed to comment on any deviation between the developer’s stated intended purpose and actual delivered app functionality that would lead to a different risk class.

After the evaluation, the results were combined and analyzed. A vote of at least 3 out of 4 reviewers was defined as a definite vote. Compliance was then assessed as ‘regulatory compliant’ if the rules applicable to the individual app were followed or as ‘regulatory non-compliant’ in the event of deviations from the applicable regulations. Apps that did not qualify as MDs, in the opinion of the experts, were always assessed as ‘regulatory compliant’, as they are not subject to any special rules within the meaning of the MDR.

#### Gamification analysis

To assess the specific risks of the apps associated with their gamified aspects, an in-depth analysis of a subset of eight apps was carried out independently by two reviewers, one professor for regulatory science and consultant for regulatory affairs (author S.G.), and one physician and game developer (author O.F.). Only apps with a majority panel decision of MD status (3 out of 4 votes) were included. Selected apps had to be available in Germany and English, free of charge, available in the Google PlayStore, and accessible without a doctor’s prescription. Apps with similar functionalities by the same developer were excluded. Each app was downloaded and tested for at least 10 min. The number of gamification elements, following the adopted definition^4^, was stated, and the primary purpose of each app was assessed. Both reviewers had to report their personal game feel on an arbitrary scale from 1 (lowest) to 5 (highest). The scale was developed by three gamification experts (O.F., K.J.F., M.H.) to determine the degree of gamification. Both assessors had to answer the question “How close is the experience, interaction and appearance of this app to a full-fledged game?” on a scale from 1 (infrequent or minimal non-immersive gamefeel through gamification elements), 2 (occasional or moderate non-immersive gamefeel through gamification elements), 3 (occasional immersive aspects of gamefeel, but separated by user experience with no or minimal game feel), 4 (frequent immersive aspects of game experience throughout), and 5 (Fully immersive game experience throughout, the app experience feeling fully like a game). The rationale behind using this self-developed scale was that existing scales make the degree of gamification dependent on the number of elements used^70^, leaving out the perceptual level^80,81^. However, since the number of elements does not necessarily correlate with the quality of gamification or the game feel^25^, we had to resort to a self-developed scale.

The specific medical risks that emerge from or are connected with the implemented gamification elements were assessed according to medical device risk assessment principles in the applicable compulsory standard ISO 14971^50^. Firstly, *hazards* (a potential source of harm), the *foreseeable sequence of events* (sequence of events or circumstances that lead to a hazardous situation), the *hazardous situation* (circumstance in which people are exposed to a hazard)*,* and the *harm* (injury or damage to the health of people) to the user were defined. The severity of harm, which is dependent on the intended use of the app, was then rated from 1 (Negligible—Inconvenience or temporary discomfort), 2 (Minor—Temporary injury or physical and mental impairment requiring simple or no professional medical intervention), 3 (Moderate—Temporary injury or physical or mental impairment requiring professional medical intervention (excluding surgical intervention)), 4 (Severe–Permanent physical or mental impairment or life-threatening injury requiring surgical intervention) to 5 (Catastrophic—User death as a direct result of hazard). The strength of the association of gamification elements with risks was reported on a scale from *none* (Gamification elements are not responsible for any part of the reported risk), *weak* (Gamification elements are minimally responsible for the reported risk, contributing insignificantly, with other aspects of the app being more responsible), *moderate* (Gamification elements are moderately responsible for the reported risk, contributing to an increase in risk likelihood or severity, with other aspects of the app being similarly responsible), to *strong* (Gamification is a major factor in the reported risk, significantly increasing its likelihood or severity, with other aspects of the app being minimally responsible).

### US assessment

One senior expert for US regulation performed the comparative US market analysis. All apps that were not available in the US app store were excluded. The definitions followed FDA guidelines^51,65,82^ and the FFDCA^32^. In the first step, an app was defined as an MD based on whether the product acts on a disease or disordered state and references the diagnosis, cure, mitigation, treatment, or disease prevention based on the app’s claim and intended use. In the second step, the specific risk class a MD would likely fall under (Class I, Class II, Class III) was based on the expert’s knowledge. Apps that have claims closer to mitigation (e.g., managing symptoms of MD) or if the product acts on a symptom rather than the disease itself (e.g., managing feelings of anxiety, where anxiety is not a disease), the device was considered as Class I. The device was considered Class II if the claims were specific to diagnosis, cure, or treatment. Since there are currently no software-only digital products in the US in Class III, such a classification was considered unlikely. In the third step, the most probable regulatory pathway was defined. Class I MDs mainly belong under the self-declared enforcement discretion status when they pose a low risk without needing evaluation or evidence^51^ or could belong in the self-declared 510(k) exempt status^52^. Class II MDs could also belong to the 510(k) exempt status, follow the 510(k) pathway if a predicate is available, or follow the De Novo pathway if no such predicate is available^82^.

## Supplementary Information

Supplementary Information.

## Data Availability

Access to the de-identified dataset of gamified mHealth-Apps can be made available by contacting the corresponding author.

## Code availability

Only publicly available libraries were used for the statistical analysis.
