# Supplementary material for: The regulatory status of health apps that employ gamification
Source: Sci Rep. 2024 Sep 9;14:21016. doi: 10.1038/s41598-024-71808-2 (PMC11383928; doi:10.1038/s41598-024-71808-2)
Supplement: Supplementary file 1 — Supplementary Information. [file 41598_2024_71808_MOESM1_ESM.pdf]

Supplemental Material - The regulatory status of health apps that employ gamification

Authors: Oscar Freyer[1,2], Kamil J. Wrona[3], Quentin de Snoeck[4,5], Moritz Hofmann[2], Tom Melvin[5], Ashley Stratton-Powell[5,6], Paul Wicks[7], Acacia C. Parks[8], Stephen Gilbert[1¶]

1- Else Kröner Fresenius Center for Digital Health, TUD Dresden University of Technology, Dresden, Germany
2- WhalesDontFly H&F GmbH, Berlin, Germany
3- Bielefeld University of Applied Sciences and Arts, Bielefeld, Germany
4- Therapixel, Nice, France
5- School of Medicine, Trinity College, University of Dublin, Dublin, Ireland
6- RQM+, Altrincham, Cheshire, UK
7- Wicks Digital Health, Advantage House, Stowe Court, Lichfield, UK
8- Liquid Amber, Cortland, OH, USA
¶- corresponding author (Professor) Stephen Gilbert: stephen.gilbert@tu-dresden.de

Supplemental Material 1

Steps to regulatory approval under EU's MDR

Regulatory approval for medical devices (MDs) under the EU's Medical Device Regulation (MDR) is a structured process designed to ensure that devices are safe and effective before being introduced to the market[1,2]. **Figure 1** provides an overview of this process. The first step is defining the intended purpose of the product and determining whether it qualifies as a MD[1]. For this, the manufacturer checks whether the device's intended purpose meets one of the definitions provided in the MDR: "Medical device means any instrument, apparatus, appliance, software (…) to be used (…) for one or more of the following specific medical purposes: diagnosis, prevention, monitoring, prediction, prognosis, treatment or alleviation of disease; diagnosis, monitoring, treatment, alleviation of, or compensation for, an injury or disability; investigation, replacement or modification of the anatomy or of a physiological or pathological process or state; (…)[1]." Additional guidelines are available to help determine whether the device qualifies as a MD[3–5].

Once established as a MD, the product must be classified according to specific risk classes[1,5]. These classes range from low risk (Class I) to high risk (Class III), with the classification influencing the complexity of the regulatory pathway, especially of the conformity assessment[1]. The risk classification is based on 22 rules defined in the MDR and explained in additional guidelines[1,5]. For MDs classified as Class I, manufacturers can self-declare their conformity with the MDR's requirements without the involvement of a Notified Body, while devices with a higher risk classification have to involve a Notified Body to evaluate the device's compliance with the relevant conformity assessment route [1,2].

Figure 1. Pathway to regulatory approval of medical devices under MDR. The flowchart is based on the MDR[1], guidance documents[4,5], and on existing literature[2]. [a]Must be certified by a notified body for MDs other than Risk class I. [b]This includes clinical investigation for certain MDs. [c]Includes an audit by a notified body for MDs other than Risk class I.

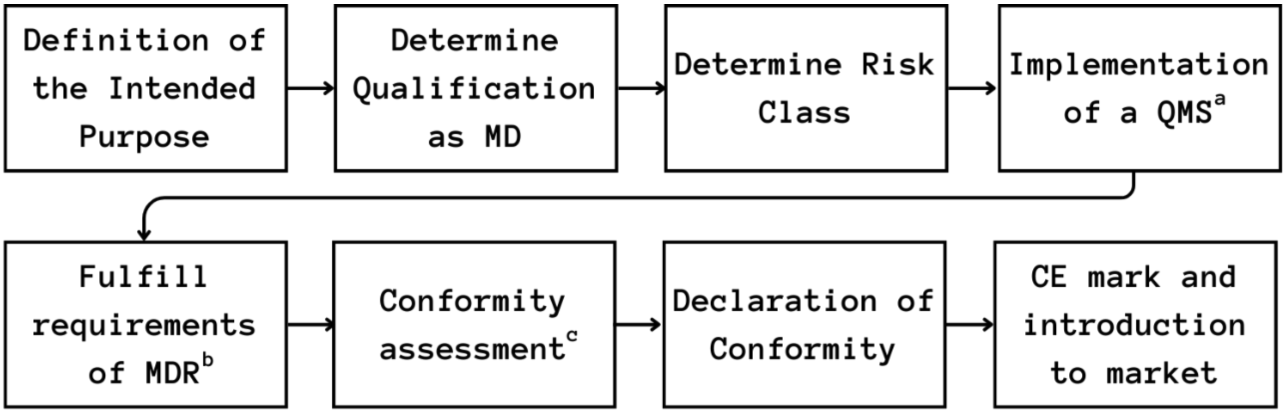

Before conformity can be declared, manufacturers must fulfill all general safety and performance requirements of the MDR by conforming to harmonized standards (e.g., ISO 14971) where applicable, and by establishing a quality management system (QMS), which must be certified by a Notified Body in case of risk classes other than Class I[1,2]. Even for Class I MDs, the most practicable option for developers for the establishment of a QMS is generally to do this according to ISO 13485, with certification by a Notified Body. In some cases, and mandatory for Risk Class III devices, a clinical investigation on humans must be conducted prior to placing the product on the market[2]. After the conformity assessment, the manufacturer declares its conformity and affixes the CE mark to their product[2].

Criteria for the risk classification under EU's MDR

After the manufacturer has assessed whether the device qualifies as a MD and falls under MDR, the risk class of the device is determined[2,5]. The intended purpose of the device and the clinical claims for the product are crucial for this. Based on these, the manufacturer needs to check which rules apply and then compare the characteristics and the intended purpose of his product with the definition provided by the applicable rules[5]. The MD then falls into the highest class provided by the applicable rules[5]. MDCG 2021- 24 provides a flowchart as guidance for manufacturers for this process and useful examples[5].

Identifying the risk classification of the MD is an integral part of the approval process. This process is meant to classify MDs based on the risk they pose for the patient into four risk classes (Class I (low risk), Class IIa, Class IIb, and Class III (high risk))[1]. The risk classification is based on 22 rules provided within the MDR, defined and explained by additional guidance documents[1,5]. The most relevant rule for SaMDs, which would include gamified health apps, is Rule 11[1,4,5]. Rule 11 states that "Software intended to provide information which is used to take decisions with diagnosis or therapeutic purposes is classified as class IIa, except if such decisions have an impact that may cause: death or an irreversible deterioration of a person's state of health, in which case it is in class III; or a serious deterioration of a person's state of health or a surgical intervention, in which case it is classified as class IIb. Software intended to monitor physiological processes is classified as class IIa,

except if it is intended for monitoring of vital physiological parameters, where the nature of variations of those parameters is such that it could result in immediate danger to the patient, in which case it is classified as Class IIb. All other software is classified as Class I[1].”

Examples of MDs in different risk classes

MDs can be classified into different risk classes based on the rules provided by the MDR[1]. Table 1 lists some examples of software and non-software devices based on MDCG guidance documents[3–5].

Table 1. Examples of medical devices in different risk classes under MDR. The table is based on examples provided in MDCG guidance documents[3–5].

	Description	Applicable Rule in MDR	Risk Class
Non Software Devices	Devices intended in general for external patient support (e.g. hospital beds, patient hoists, walking aids, wheelchairs, stretchers, dental patient chairs)	1	I
	Stethoscopes	1	I
	Devices intended to be used as channels in active drug delivery systems, e.g. tubing intended for use with an infusion pump	2	IIa
	Wound dressings intended for ulcerated wounds having breached the dermis	4	IIb
	Tracheal tubes	5	IIa
	Heart valve occluders, sizers and holders	6	III
	Prosthetic heart valves	8	III
	Dermatoscopes with integrated light sources	10	I
	Magnetic resonance equipment	10	IIa
Software Devices	Cognitive therapy medical device software (MDSW) where a specialist determines the necessary cognitive therapy based on the outcome provided by the MDSW.	11	IIa
	MDSW intended to perform diagnosis by means of image analysis for making treatment decisions in patients with acute stroke.	11	III
	A mobile app intended to analyse a user’s heartbeat, detect abnormalities and inform a physician accordingly. MDSW intended for diagnosing depression based on a score resulting from inputted data on patient symptoms (e.g. anxiety, sleep patterns, stress etc.).	11	IIb
	Devices intended to be used to obtain readings of vital physiological signals in routine check-ups including monitoring at home.	11	IIa
	MDSW app intended to support conception by calculating the user’s fertility status based on a validated statistical algorithm.	11	I
	Hospital Information Systems	-	Non-MD
	Communication systems	-	Non-MD
	Medical Calculator (e.g., for CHA_2DS_2-VASc Score)	11	IIa or higher

References

1. European Parliament, European Council. Regulation (EU) 2017/745 of the European Parliament and of the Council of 5 April 2017 on Medical Devices, Amending Directive 2001/83/EC, Regulation (EC) No 178/2002 and Regulation (EC) No 1223/2009 and Repealing Council Directives 90/385/EEC and 93/42/EEC (Text with EEA Relevance)Text with EEA Relevance. (2017).

2. Keutzer, L. & Simonsson, U. S. Medical Device Apps: An Introduction to Regulatory Affairs for Developers. JMIR MHealth UHealth **8**, e17567 (2020).

3. European Comission. Manual on Borderline and Classification under Regulations (EU) 2017/745 and 2017/746 - Version2 - December 2022.

4. Medical Device Coordination Group (MDCG). MDCG 2019-11 Guidance on Qualification and Classification of Software in Regulation (EU) 2017/745 – MDR and Regulation (EU) 2017/746 – IVDR. (2019).

5. Medical Device Coordination Group (MDCG). MDCG 2021-24 Guidance on Classification of Medical Devices - October 2021. (2021).
